# Spherezymes: A novel structured self-immobilisation enzyme technology

**DOI:** 10.1186/1472-6750-8-8

**Published:** 2008-01-31

**Authors:** Dean Brady, Justin Jordaan, Clinton Simpson, Avashnee Chetty, Cherise Arumugam, Francis S Moolman

**Affiliations:** 1 CSIR Biosciences, Ardeer Road, Modderfontein, 1645 South Africa; 2 CSIR Materials Science and Manufacturing, Meiring Naudé Road, Brummeria, Pretoria, 0001 South Africa

## Abstract

**Background:**

Enzymes have found extensive and growing application in the field of chemical organic synthesis and resolution of chiral intermediates. In order to stabilise the enzymes and to facilitate their recovery and recycle, they are frequently immobilised. However, immobilisation onto solid supports greatly reduces the volumetric and specific activity of the biocatalysts. An alternative is to form self-immobilised enzyme particles.

**Results:**

Through addition of protein cross-linking agents to a water-in-oil emulsion of an aqueous enzyme solution, structured self-immobilised spherical enzyme particles of *Pseudomonas fluorescens *lipase were formed. The particles could be recovered from the emulsion, and activity in aqueous and organic solvents was successfully demonstrated. Preliminary data indicates that the lipase tended to collect at the interface.

**Conclusion:**

The immobilised particles provide a number of advantages. The individual spherical particles had a diameter of between 0.5–10 μm, but tended to form aggregates with an average particle volume distribution of 100 μm. The size could be controlled through addition of surfactant and variations in protein concentration. The particles were robust enough to be recovered by centrifugation and filtration, and to be recycled for further reactions. They present lipase enzymes with the active sites selectively orientated towards the exterior of the particle. Co-immobilisation with other enzymes, or other proteins such as albumin, was also demonstrated. Moreover, higher activity for small ester molecules could be achieved by the immobilised enzyme particles than for free enzyme, presumably because the lipase conformation required for catalysis had been locked in place during immobilisation. The immobilised enzymes also demonstrated superior activity in organic solvent compared to the original free enzyme. This type of self-immobilised enzyme particle has been named spherezymes.

## Background

Enzymes are widely used in industrial processes, from environmental applications such as effluent treatment and bioremediation, through food and beverage processing, to fine chemical processes such as stereoselective biocatalysis [1,2]. This broad application is possible because enzymes provide highly selective catalysis [3,4]. Furthermore, the use of enzymes in catalysis potentially reduces toxic or wasteful by-products, effluent load and energy consumption (due to milder reaction conditions), thus providing an environmentally efficient alternative to many currently available chemical catalysts [5,6].

However the application of biocatalysts can suffer from several general drawbacks. In addition to the often relatively high cost compared to current chemical catalysts, enzymes may display instability towards temperature, pH, solvents, oxidation and shear, resulting in limited suitability or shelf life. Moreover, soluble enzymes cannot be easily recovered from aqueous media and hence often cannot be reused. Thus, although enzymes such as lipases in particular have found extensive application in organic synthesis [7], effective stabilisation remains a technical hurdle limiting use of enzymes such as *Pseudomonas *lipases in industrial-scale biotransformations [8].

To overcome some of these problems enzymes are frequently immobilised, primarily by attachment onto supports. Immobilisation has several advantages including effective recovery and recycle through centrifugation or filtration and improved enzyme stability [9-11]. However, conventional support-based immobilisation techniques involve the use of costly resins, and is usually associated with a large loss in enzyme activity compared to the native enzyme, as well as a reduction in the specific and volumetric activity of the biocatalyst to approximately 10% due the limited loading capacity of these supports [9]. An alternative to immobilisation onto supports is self-immobilisation through cross-linking of the enzyme. This negates the need for an immobilisation matrix, thereby reducing the cost, and increasing specific and volumetric activity.

Currently there are a number of published or patented methods for self-immobilised biocatalysts, of which the most relevant are Cross-Linked Enzyme Aggregates (CLEA, [12]), Cross-Linked Spray Dried Enzyme (CSDE, [13]), Cross-Linked Enzyme from Solution [14,15], and Cross-Linked Enzyme Crystals (CLEC, [16]). In particular CLEC, and more recently CLEA, have shown application in biocatalysis [17,12]. However, the CLEC technology requires enzyme of high purity which is also easily crystallisable, and therefore has been relatively expensive and limited in range, which has adversely affected its commercial applicability. The alternative CLEA process depends on initial precipitation of the enzyme, followed by cross-linking. This is a less expensive and relatively simple process that concomitantly purifies the enzyme to a degree during the partially selective precipitation step. However, both CLEA and CLEC technology may result in diffusion constraints with increasing particle size [18].

We have discovered that it is possible to self-immobilise enzymes in an emulsion while retaining activity [19]. This relatively inexpensive method allows for formation of spherical catalytic macro-particles (spherezymes) that would be of interest to synthetic chemists. Moreover the method permits size control and incorporation of macro-structure into the self-immobilised particle during the immobilisation process. This could provide advantages for control of substrate-enzyme interaction, perhaps improving overall activity.

**Figure 1:**
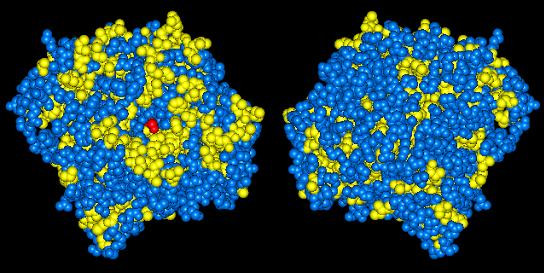
*Candida rugosa *lipase 1 top and bottom views as visualized in Accelerys ViewerLite 4.2. Yellow indicates hydrophobic amino acids, blue all other amino acids, and red the active site. Note the hydrophobic ring around the active site. Coordinates were obtained from the RCSB Protein Data Bank [21].

**Figure 2:**
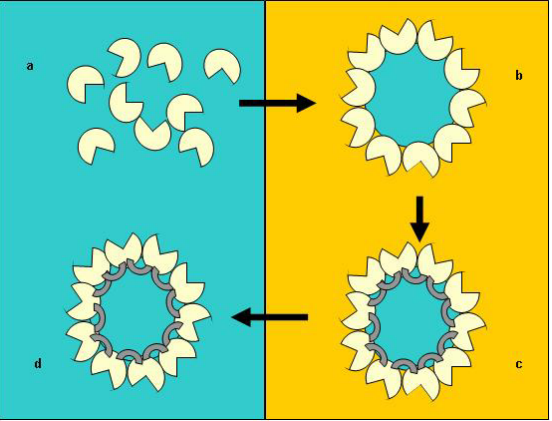
Lipase spherezymes immobilisation method. Lipase (a) in aqueous medium (left) is added to an excess of a hydrophobic solvent (right), forming a water-in-oil emulsion (b), wherein the lipase migrates to the phase boundary and orientates the hydrophobic face associated with the active site towards the hydrophobic layer. The enzyme is subsequently cross-linked (grey crescents) to make the structure permanent (c) and the solvent removed (d).

The enzyme sub-class of lipase [EC 3.1.1.3] was chosen as the initial enzyme for investigations due to its biocatalytic importance for chiral resolution of chemicals and pharmaceutical intermediates. Lipases typically possess a hydrophobic surface, often containing a 'lid' region associated with the active site [20] (Fig. 1), and it is believed that this surface associates with the hydrophobic phase at the interface between hydrophilic and hydrophobic fluids. Within a water-in-oil emulsion, these proteins would therefore tend to localise and orientate at the spherical interface of the emulsion droplet. Subsequent cross-linking of the protein would then form a self immobilised particle (Fig. 2).

The aim of the current research was to explore the concept of emulsion based immobilisation of enzymes, to determine whether it was capable of providing suitable catalysts, and to investigate some of the characteristics regarding the inclusion of structure into these self-immobilised enzyme particles.

## Results

### Visualisation

**Figure 3:**
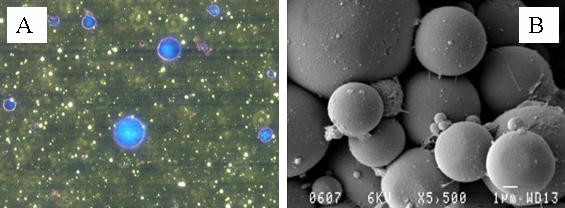
Optical microscope photographs of spheres stained with comassie blue (left) and scanning electron microscope image (right) of 20% lipase/80% albumin spheres.

Lipase and bovine serum albumin (BSA) were immobilised using glutaraldehyde in a water-in-mineral oil emulsion with nonoxynol-4 as the surfactant. As anticipated, optical and scanning electron microscopy analysis of the spherezymes revealed spherical particles (Fig. 3), as would be associated with protein immobilisation within emulsion droplets.

### Particle size

**Figure 4:**
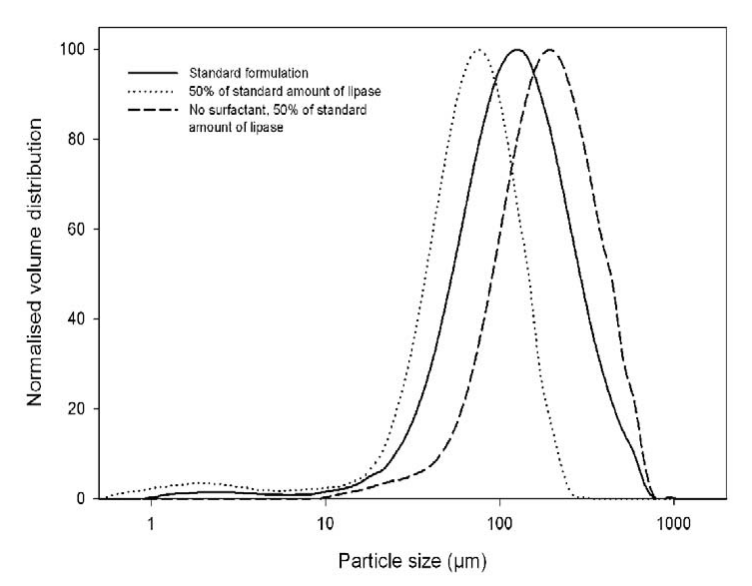
Size distribution of lipase spheres made with surfactant in mineral oil as determined by LLS. Standard formulation method (_____), 50% lipase concentration (.........), 50% lipase concentration and surfactant omitted (-------).

From the SEM images, the size of the individual spherical particles were determined to be between 0.5–10 μm in diameter (Fig. 3b). However from laser light scattering experiments it appears that the particles aggregate with a size distribution between 10 and 100 μm. Using the current standard procedure for spherezyme formation the average particle size diameter was 62.0 μm, (Sauter mean diameter d_32 _= 62.0 μm, span = 1.96, Fig. 4).

The size of both the individual sphere particles and the aggregate particles could be controlled by manipulating the process conditions. Upon halving the lipase concentration, the particle size distribution shifted to smaller particle sizes (d_32 _= 24.8 μm, span = 1.36). When both the lipase amount was halved and surfactant omitted, the distribution shifted to larger particle sizes (d_32 _= 137.1 μm, span = 1.61). As means of separation during application, enzyme activity could be retained without loss by filtration on typical biological filters (0.22 μm).

Aggregate formation could be due to agglomeration through low charge (zeta potential), hydrophobic interaction, or through secondary cross-linking of the spheres through residual free aldehydes from the cross-linking process. As low zeta potential can result in agglomeration (as a function of aggregation), the zeta potential of an emulsion containing albumin prior to crosslinking was measured. As a rule of thumb, particles having zeta potential values greater than ± 30 mV tend to be more stable, while conversely values smaller than ± 30 mV indicate a tendency to agglomerate [22]. The average zeta potential obtained for albumin spheres was -45 ± 1.6 mV, with n = 3, indicating a low tendency for agglomeration. Particle agglomeration may also be reduced through steric hindrance/repulsion (typically this entails the use of a polymeric surfactant). The aggregates could be dispersed through mild sonication. As albumin is hydrophilic, this would suggest that the aggregates are formed by covalent cross-linking. Post-treatment with amino acids appears to reduce this effect [19].

### Yield

The mass yield of lipase incorporation into the spherezymes was 100.2% ± 7.9% SD. This suggests that enzyme incorporation was near complete.

### Specific density

The enzyme particles had a density between 1.11 and 1.23 g.ml^-1 ^according to their buoyancy in salt solutions. As a standard separation technique, activity could be fully recovered by centrifugation at 3000 × g on standard bench-top centrifuges.

### Effects of cross-linking method on activity

Initial experiments with glutaraldehyde treatment of lipases in an emulsion resulted in large loss of activity (approximately 99%). However later it was discovered that the lipases could be protected in some cases against glutaraldehyde deactivation by the use of a 'protectant', typically a substrate, which is proposed to occupy the active site during the cross-linking process. Addition of 10 μl (0.2% v/v) tributyin to the reaction mixture increased the activity of *P. fluorescens *lipase on *p*-nitrophenyl butyrate 202 fold, from less than 1 percent to 125% of original free lipase activity (0.101 ± 0.002 to 20.4 ± 0.631 μmol.min^-1^.mg^-1^). Optimisation of the ratio of immobilisation reagents provided a further tenfold improvement in activity on *p*-nitrophenyl butyrate.

Changing to a combination of glutaraldehyde and ethylenediamine (EDA) as a cross-linker [23] provided benefits. After 12 hours protein cross-linking with 30 μl of pre-mixed glutaraldehyde EDA cross-linker, the *p*-nitrophenyl palmitate activity increased from 386 to 2184 μmol.min^-1^.mg^-1 ^and *p*-nitrophenyl butyrate activity from 202 to 345 μmol.min^-1^.mg^-1 ^compared to glutaraldehyde alone, indicating improved access of the substrate to the enzyme, possibly due to the formation of longer cross-linking chains compared to glutaraldehyde cross-linking.

The reproducibility of the method is important for possible application of this technique. Five batches of *P. fluorescens *lipase spherezymes were produced. Against the smaller *p*-nitrophenyl butyrate substrate the activity of the spheres was 159% ± 30.3% of that of the free enzyme for fourteen samples with a standard deviation of ± 30.0% between batches. This increase in activity may be attributed to hyper-activation of the enzyme, probably due to the locking open of the lipase active site lid or other modification of conformation during the immobilisation process.

Apart from the *P. fluorescens *lipase, the method was successfully applied to lipase from *Candida. rugosa*, *Rhizopus. oryzae *as well as wheat-germ lipase. Wheat-germ lipase, *R. oryzae *and *C. rugosa *lipase spherezymes maintained 40%, 18% and 54% activity respectively on *p*-nitrophenyl butyrate as the substrate.

### Biocatalyst recycle

**Figure 5:**
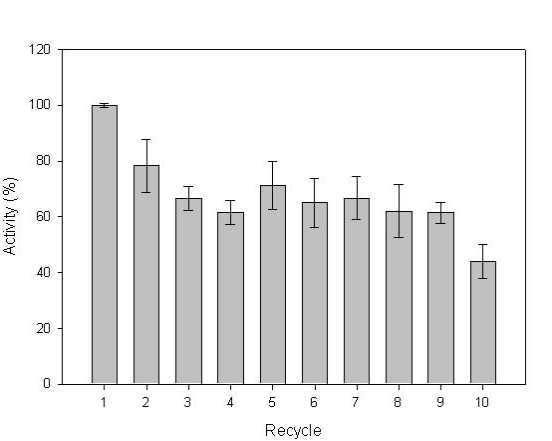
Results achieved for Denilite^® ^laccase spherezymes during ten recycles with pyrogallol as the substrate.

As enzymes may be expensive, re-use of the catalyst is often a necessity. The *P. fluorescens *lipase particles were recycled 6 times with washing by centrifugation with no observable loss of activity on *p*-nitrophenyl butyrate. Denilite^® ^laccase spherezymes were also investigated, using pyrogallol as a substrate. The spheres were reacted ten times with pyrogallol with recovery and washing between each reaction. Laccase activity after these ten recycles retained significant activity compared to the original activity of the spheres (Fig. 5).

### Combined protein particles

**Figure 6:**
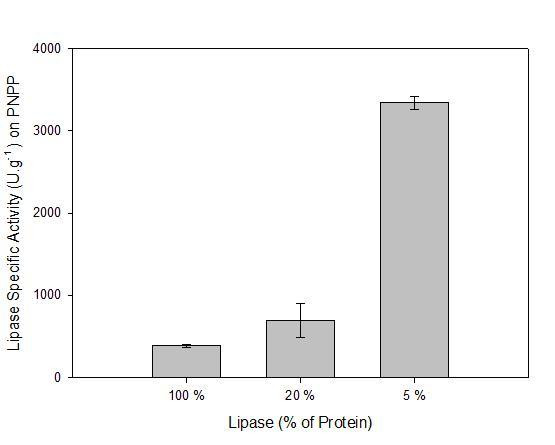
Spherezyme particles consisting of various percentage combinations of *P. fluorescens *lipase and BSA. Lipase specific activity (on *p*-nitrophenyl palmitate) is represented as a function of lipase mass as opposed to total protein mass. Data was generated from particles that were formed using 40 μl glutaraldehyde-EDA mix as a cross-linker and nonoxynol-4 as the surfactant.

The immobilisation of multiple proteins in single spherezymes was investigated. The results of including BSA in combination with *P. fluorescens *lipase provided a strong indication of the predicted selective migration of lipase to the phase interface. The difference in molecular mass of the two *p*-nitrophenyl esters used to assay activity permitted evaluation of the mass transfer properties of the spheres based on substrate size. An important observation was that although absolute specific activity decreased with increasing percentage BSA inclusion in the particles (as would be expected), the lipase specific activity (calculated solely on lipase mass) increased for *p*-nitrophenyl palmitate (Fig. 6). This was not observed in the case of *p*-nitrophenyl-butyrate (data not shown), indicating that it was specifically a surface related phenomenon.

**Figure 7:**
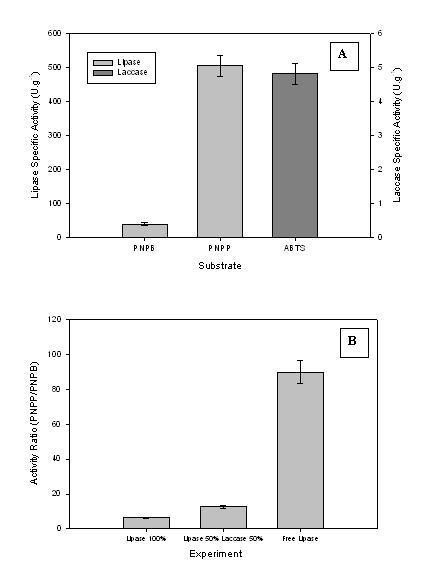
Activity of a spherezyme particles comprised of a combination of enzymes (*P. fluorescens *lipase and Denilite^® ^laccase) cross-linked with glutaraldehyde: PNPB – *p*-nitrophenyl butyrate; PNPP – *p*-nitrophenyl palmitate (A). The ratio of *p*-nitrophenyl palmitate to *p*-nitrophenyl butyrate activity of these combined enzyme particles can be compared to free *P. fluorescens *lipase and lipase spherezyme particles (B).

Combination immobilised enzyme particles were formed using co-immobilised *P. fluorescens *lipase and Denilite^® ^laccase (Fig. 7A), and were able to catalyse both hydrolysis and oxidation reactions. The ratio of *p*-nitrophenyl palmitate to *p*-nitrophenyl butyrate was used as an indication of surface to volumetric activity and hence distribution of enzyme within the particle. Similar to the data where lipase was combined with BSA, it appears that the lipase selectively migrated to the exterior of the particle since in the dual enzyme particles (laccase and lipase) there was a doubling of the ratio of *p*-nitrophenyl palmitate to *p*-nitrophenyl butyrate activity compared to the lipase particles (Fig. 7B).

### Activity in organic solvents

**Figure 8:**
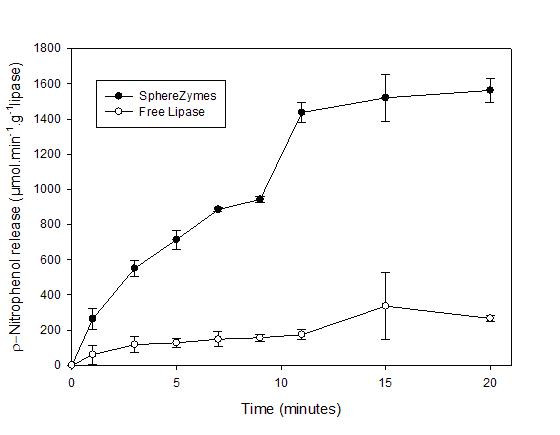
*p*-Nitrophenyl palmitate hydrolysis in water saturated *n*-heptane using non-immobilised and spherezyme *P. fluorescens *lipase.

The application of enzymes, and particularly lipases, in organic solvents is quite common in biocatalysis, and hence a new immobilisation method should preferably provide an organic solvent-tolerant catalyst. The *p*-nitrophenyl palmitate hydrolysis in water-saturated *n*-heptane [24] using spherezymes prepared from *P. fluorescens *lipase was compared to non-immobilised enzyme based on equivalent mass. The hydrolytic activity of spherezymes lipase on *p*-nitrophenyl palmitate was calculated to be 5.9 fold higher than that of non-immobilised lipase in water saturated *n*-heptane (Fig. 8).

**Figure 9:**
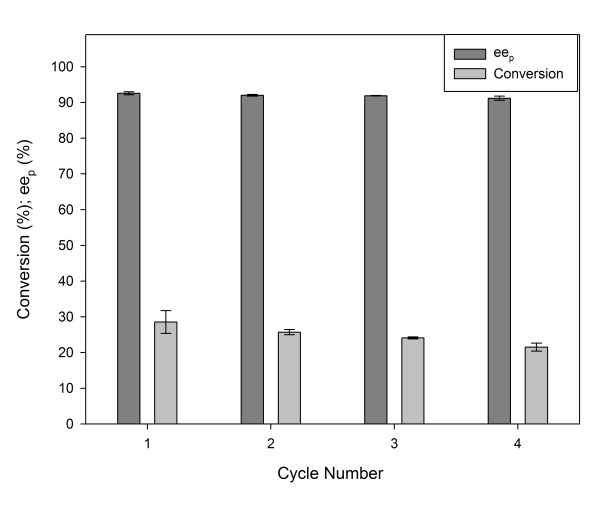
Selective esterification of (-)-menthol by vinyl acetate in *n*-heptane using *P. fluorescens *lipase spherezyme as a catalyst.

To evaluate the synthetic capacity of *P. fluorescens *lipase spherezymes, they were used to esterify (+,-)-menthol present in a diastereomer mixture (four pairs of racemic diastereomers; menthol, isomenthol, neomenthol, and neoisomenthol) that had been generated from hydrogenation of thymol. Four sequential reactions were performed, each for 24 hour reaction times, and an average conversion of 25.0% ± 3.03 menthol to (-)-menthyl acetate was obtained with a consistent ee_p _of 91.9 ± 0.599 (Fig 9), which is similar to the results achieved by Chaplin *et al *(2002) [25]. An additional recycle was performed to determine the ee_p _at near maximum conversion, where the incubation time was extended to 94 hours, and this provided a conversion of 49.4% ± 0.35 and an ee_p _of 87.1 ± 0.30 on the menthol racemate.

### Effects of lyophilisation

In order to preserve the activity and shelf-life of the spherezymes, lyophilisation was investigated as a possible preservation technique. *P. fluorescens *lipase spherezymes were lyophilised, and the effect of the lyo-protectant 5% m/v mannitol on enzyme activity was assessed. The particles, lyophilised in the presence of protectant maintained 101.4% residual activity against *p*-nitrophenyl butyrate. Without protectant only 17.2% activity was maintained.

## Discussion

Self-immobilised enzymes (such as CLEAs and CLECs) provide 10–1000 times higher volumetric activity than carrier bound immobilised enzymes [18], providing significant potential advantages for commercial reactions.

These initial experiments have shown that enzymes can be self-immobilised as catalytically active spherical bodies using an emulsion based technique in the presence of surfactants and active site protectants. The technology was successfully applied to four lipases and a laccase. Co-immobilisation of lipase with other proteins, BSA or laccase, was also shown. The ability of the biocatalyst to be recovered by centrifugation and recycled was also demonstrated using both enzyme classes immobilised with this technology.

The technique also permits control of particle size, and hence surface area. The control of surface area to volume allows for optimisation of reactant diffusion. Interestingly a modification of the CLEA process has recently been published [26] in which an emulsion based technique is used to control particle size, although in his case the emulsion is broken during the immobilisation process, and the resultant product forms fibrous aggregates rather than spheres.

The need for addition of surfactants is moderated by the surfactant properties of the enzyme itself, particularly in the case of proteins possessing surfactant properties. For example, in the current results the reduction in particle size when lipase amount was decreased is possibly due to the lipase surfactant activity. Excessive surfactant addition can cause bridging flocculation, leading to agglomerates, thus resulting in apparent larger average particle size during analysis [27]. This indicates that the level of surfactant activity of protein utilized in spherezyme manufacture should be determined and taken into account when formulating the emulsions. When the surfactant was removed, the size distribution increased as would be expected.

The spheres had a particle size of 0.5–10 μm, and formed aggregates of 1 – 1000 μm, with a mean of 62 μm. We suggest that this size range would be an ideal compromise of surface area to recoverability by filtration or centrifugation. Adjustments to the particle size range can be made through controlling surfactant type and concentration in combination with agitation rate and method. The density of the particles was only slightly greater than water, while the Zeta-potential of the spherezymes was -45 ± 1.6 mV, indicating low tendency for flocculation or agglomeration, and hence the particles remained suspended in the reaction medium with only mild agitation.

Mass yield determinations suggest an approximate 100% mass recovery. We have also optimised immobilisation methods to achieve greater than 100% enzyme activity yields for *P. fluorescens *lipase spherezymes compared to the native lipase, as determined by release of *p*-nitrophenol from *p*-nitrophenyl butyrate. Many lipases, such as *Pseudomonas *and *Candida rugosa *lipases have "lids" in the region of the active site [20]. These lids are opened in the presence of hydrophobic interfaces, thereby activating the enzyme [28]. The observed hyperactivation may be due to the fixed opening of the lipase "lid" or other structural modifications. Immobilised lipase activity exceeding that of the native lipase has also been observed using the CLEA technique [12]. In addition, we have shown that the surface properties of the spheres could be modified by attachment of amines, such as the hydrophobic phenylglycine, further modifying the hydrophobic properties of the enzyme [19].

Preliminary experiments were performed using multiple proteins to demonstrate inclusion of structure during immobilisation. Initially albumin was selected due to its hydrophilic nature and relatively large number of lysine residues with approximately three times that of lipases (Swiss-Prot entries P02769, P26504, P20261 for BSA, *P. fluorescens *lipase A and *C. rugosa *lipase 1 respectively) which would aid in cross-linking. It was anticipated that the lipases would migrate to the phase interface and orientate due to the hydrophobic surface prior to cross-linking. This feature was also investigated by co-immobilisation of laccase and lipase, where conversely the laccase tends to possess fewer lysine residues (Swiss-Prot entries Q99044 and Q12718). The more hydrophobic of the two proteins (lipase) appeared to migrate to the surface of the emulsion droplets. Where combination enzymes are involved, such a structure may be used to catalyse multiple reactions. Moreover, an external protein or enzyme may protect an internal enzyme from inactivation, such as by gas bubbles [11].

The immobilisation of enzymes tends to make them more stable in unusual environments. For example improved lipase (*Pseudomonas alcaligenes *lipase) activity in organic solvent has been observed after immobilisation in some instances using the CLEA technique [12]. The activity of lipase spherezymes in *n*-heptane demonstrated that the self-immobilised particles are better suited to working in *n*-heptane than that of the non-immobilised enzyme, which tends to clump in organic solvent. As the immobilised particles are generated in a water in oil emulsion, it is therefore likely that active biocatalysts successfully produced by this method will be comparatively stable in organic solvents.

## Conclusion

We have successfully demonstrated the concept of creating structured self-immobilised spherical enzyme particles through the use of an emulsion based process. They are relatively inexpensive to manufacture, and sufficiently robust to retain activity after centrifugation or filtration for recycling, and active in both in aqueous and organic media. The capacity to orientate the enzymes either towards or away from the interface (phase boundary) and the ability to manipulate the radial location of component proteins when immobilising two or more proteins could also allow for optimising activity, especially for large substrates. This technique allows for the incorporation of fine structure into immobilised enzyme bodies, allowing for the generation of more sophisticated biocatalysts.

## Methods

### Enzymes and proteins

*Pseudomonas fluorescens *lipase (Amano AK) was obtained from Amano. Denilite^® ^laccase was a kind gift from Novozymes. *Candida rugosa *lipase was kindly donated by Altus Biologics (USA). Wheat-germ lipase, and *Rhizopus oryzae *lipase were purchased from Sigma-Aldrich (Fluka). Bovine serum albumin (BSA) Fraction V, was obtained from Sigma-Aldrich.

Ultra-filtration or dialysis of the enzyme solutions was performed prior to use as salts and additives in the preparations may inhibit emulsion formation or cross-linking. Lipase in Tris-Cl Buffer (20 mM, pH 8.0) was filtered through an Amicon ultrafiltration unit fitted with a 10 K polyethersulfone membrane (Omega – Pall) at 45 psi. The ultrafiltered enzyme was then washed with 2 volumes of Tris-Cl Buffer (20 mM, pH 8.0) and followed by 1 volume of deionised water before use.

### Lipase and esterase hydrolysis activity assay

The activity of lipase based spherezymes involved the hydrolysis of *p*-nitrophenyl esters (*p*-nitrophenyl palmitate and *p*-nitrophenyl butyrate) to *p*-nitrophenol and an aliphatic carboxylic acid. The release of *p*-nitrophenol yields a yellow colour which was measured spectrophotometrically.

Lipase assay solution: Reagent A consisted of 0.0667 g of Gum arabic (Acacia tree) dissolved in Tris-Cl buffer (12 ml, 250 mM) containing 48 ml of distilled water. Thereafter 0.267 g of sodium deoxycholate was added and dissolved in the solution. Reagent B consisted of either 24 mg of *p*-nitrophenyl palmitate or 14 μl of *p*-nitrophenyl butyrate dissolved in 8 ml propan-2-ol at 37°C. Reagent B (1 ml) was added to 9 ml of reagent A with rapid stirring for approximately 20 seconds [29,30].

Enzyme solution (37.5 μl) was added to 900 μl of the lipase assay reagent and measured for activity at 410 nm with a DU800 UV-vis spectrophotometer (Beckman-Coulter) fitted with a Peltier temperature controller set at 25°C. Activity was measured at 10 second intervals over 2 minutes. The molar extinction coefficient of *p*-nitrophenol at pH 8.0 was calculated as 15.1 M^-1^.cm^-1^. One unit of enzyme activity was defined as the quantity of enzyme required to liberate one μmole of *p*-nitrophenol per minute under the above conditions.

### Laccase activity assay

Laccase assays were performed using 1 mM pyrogallol as the substrate in 100 mM succinate-lactate buffer pH 4.5. The formation of the chromophore was followed kinetically at 300 nm using a DU800 spectrophotometer (Beckman-Coulter). Alternatively 0.2 mM 2,2'-Azino-bis(3-ethylbenzothiazoline-6-sulfonic acid) diammonium salt (ABTS) in 100 mM succinate-lactate buffer pH 4.5 was used as the substrate [31,32]. The absorbance of the solution was measured spectrophotometrically at 420 nm.

### Particle size analysis

Particle size distribution analysis was carried out using a Malvern Mastersizer 2000 multiple-angle laser light scattering analyser (Malvern Instruments, United Kingdom). Particles were dispersed in water and sonicated before measurement at 25°C.

### SEM procedure

Scanning electron microscopy (SEM) preparation involved fixating the samples in a 0.1 M sodium cacodylate (Sigma-Aldrich) buffer solution containing 2.5% glutaraldehyde and 2.5% formaldehyde and filtering onto a 0.22 μm membrane (Millipore). The mixture was left to stand for 1 hour, and thereafter rinsed for 15 minutes with the buffer. The samples were then fixed with a 0.5% aqueous osmium tetroxide solution and thereafter rinsed with buffer for 15 minutes. Samples were then dehydrated with a series of ethanol solutions (30%,50%,70%, 90%) followed by three washes in 100% ethanol for 5 minutes each. Finally samples were critical point dried, mounted on stubs and sputter coated with gold before viewing using a JEOL JSM-840 scanning electron microscope.

### Mass yield determination

Triplicate 1 litre experiments were carried out using 100% albumin as the test protein. The spherezymes were prepared according to the method as described below, and then dried for 24 hours at 80°C or until weight loss could no longer be detected. Samples were cooled under desiccating conditions and weighed. The yield was expressed as a percentage of the mass of enzyme included in the emulsion. The contribution of the mass of the cross-linker and surfactant was considered negligible compared to the enzyme and therefore not compensated for in the calculation.

### Specific density

This was estimated by observing settling or flotation of particles in salt (potassium carbonate) solutions of varying densities. The spherezymes particles were suspended in K_2_CO_3 _solutions of five different densities (1.1 to 1.5 g.ml^-1^) for an hour.

### Zetapotential

To determine the electrostatic agglomeration tendency of the spherezymes, the zeta potential (or surface charge) of the spheres was determined in triplicate using the NanoZS Zetasizer, Malvern Instruments (United Kingdom).

### Method for spherezyme formation

The standard practice using *P. fluorescens *lipase is described below for a water in-oil emulsion (Fig. 2). The aqueous phase consisted of 0.18 ml of 100 mg.ml^-1 ^ultrafiltered enzyme added to 0.02 ml Tris-HCl buffer. The enzyme preparation typically had an activity of approximately 200 and 18000 μmol.min^-1^.mg^-1 ^on *p*-nitrophenyl butyrate and *p*-nitrophenyl palmitate respectively. An active site protectant (50 μL tributyrin, Sigma-Aldrich) was added to the aqueous phase prior to emulsification (final concentration of 25% v/v of initial enzyme preparation). Surfactant, 50 μL Nonoxynol-4 (ICI South Africa) or Span 20 (sorbitan monolaurate, Sigma-Aldrich), was added to the aqueous phase to a final concentration of 25% v/v of initial enzyme preparation. The aqueous phase was added to the oil phase (5 ml of mineral oil, a ratio of 40:1 to the aqueous phase). The final solution was stirred for 1 minute with magnetic stirring at 1500 rpm to emulsify. Glutaraldehyde (30 μL of a 25% solution, ACROS Organics) was added to a final concentration of 16.7% v/v of initial enzyme preparation immediately prior to emulsification. The solution was stirred for another minute at 1000 rpm and left to stand for a further 2 hours.

Subsequently the emulsion was centrifuged at 3000 × g for 15 minutes to separate the spheres. The oil phase was removed and the spheres were washed three times with 10 ml of Tris-Cl buffer (20 mM, pH 8.0). The pellet was thoroughly resuspended between each wash and recovered by centrifugation.

### Crosslinking agents

#### Glutaraldehyde

Glutaraldehyde was routinely used as the crosslinking agent, since the cross-linking capability of glutaraldehyde is well characterised in literature [33].

#### Glutaraldehyde: (EDA)

EDA (Sigma-Aldrich) was used to increase the chain length by forming glutaraldehyde-EDA chains (1:1 volume ratio of 0.33 M EDA (pH 6.0, adjusted with 6 M HCl) to 25% glutaraldehyde (aqueous solution) [30]. The glutaraldehyde and EDA were allowed to react for 15 minutes prior to the addition to protein. The amount of EDA-modified glutaraldehyde used was varied from 20 – 30 μl, depending on the protein type, quantity, and purity.

### Oil phase

To determine the effect of the oil phase on sphere formation and enzyme activity, vegetable oil and mineral oil were investigated. Since improved lipase activity was observed in spheres made with vegetable oil, it was realised that the vegetable oil had a protective property for the enzyme, perhaps by occupying the active site during cross-linking. A substrate was therefore added to the aqueous phase based on this discovery for subsequent enzyme immobilisations. Tributyrin was used as a protectant for the *P. fluorescens *lipase.

### *Pseudomonas fluorescens *lipase spherezyme activity in organic solvent

#### PNPP hydrolysis

*P. fluorescens *lipase preparations and the derived spherezymes were lyophilised prior to use. The reaction rate of lipase in organic solvent was determined according to [24] using the lipase hydrolysis assay (above) with minor modifications. Reactions were performed in duplicate in 1.5 ml brown (solvent tolerant) Eppendorff tubes containing 9 units of lipase, corresponding to 2 mg.ml^-1 ^and 1 mg.ml^-1 ^of the non-immobilised and immobilised freeze dried preparations respectively. The reactions were initiated by the addition of 1 ml water saturated *n*-heptane containing 50 mM *p*-nitrophenyl palmitate as the substrate. The reactions were incubated at 25°C with 1000 rpm reciprocal shaking with a TS-100 Thermo shaker (BOECO, Germany). Samples (50 μl of clear supernatant) were periodically removed after gentle centrifugation in a BOECO combispin for 30 seconds and added to 1 ml of 10 mM Tris-HCl buffer (pH 8) with shaking for 30 seconds followed by removal of the *n*-heptane layer. The absorbance of the aqueous layers was determined at 410 nm against a control reaction blank (no enzyme included).

#### Menthol esterification

To evaluate the activity and stability of lipase spherezymes in an industrially relevant reaction in organic solvent, the esterification of menthol [25] was performed using *P fluorescens *(Amano AK) lipase freeze dried spherezymes. A modification to the procedure for the manufacture of spherezymes was used, namely tributyrin was replaced with the menthol diastereomeric mix (50 μl) as the active site protectant during cross-linking. Biotransformations (1.5 ml) were performed in anhydrous *n*-heptane (29.5% m/v) using vinyl acetate (16.4% m/v) as the acyl donor to esterify a menthol diastereomeric mixture (42.6% m/v, derived from hydrogenation of thymol) over 24 hours. Approximately 60% of the mixture was a menthol racemate. The reactions were performed in duplicate with 10 mg of lyophilised spherezyme preparation, and incubated in a thermoshaker (Boeco TS-100) at 50°C with 1100 rpm shaking. After 24 hours the reactions were stopped and centrifuged for 1 minute at 700 × g (Boeco Combi-Spin). The sample, 20 μl, was diluted 50 fold with *n*-hexane and analysed by gas chromatography according to the method of Chaplin *et al*., 2002 [25]. The spherezymes were recycled by washing with *n*-heptane (1.5 ml) and the reactions were re-initiated by the addition of fresh substrate in *n*-heptane. The spherezymes were used in five sequential reactions.

## Abbreviations

BSA bovine serum albumin

CLEA Cross-Linked Enzyme Aggregates

CLEC Cross-Linked Enzyme Crystals

CSDE Cross-Linked Spray Dried Enzyme

EDA Ethylenediamine

PNPB *p*-nitrophenyl butyrate

PNPP *p*-nitrophenyl palmitate

SEM Scanning electron microscopy

## Authors' contributions

JJ: Principle Investigator and team leader, concepts, laboratory work. DB: Initiated research, concepts, laboratory work, research group leader, primary author of manuscript. FSM: emulsion technology concepts, competency area manager, concepts. CS: biocatalysis reactions in organic solvents. AC: SEM, zeta potential. CA: stability experiments, sphere density.

## References

[B1] van Beilen JB, Li Z (2002). Enzyme technology: an overview. Curr Opin Biotechnol.

[B2] Panke S, Held M, Wubbolts M (2004). Trends and innovations in industrial biocatalysis for the production of fine chemicals. Curr Opin Biotechnol.

[B3] Patel RN (2006). Biocatalysis: synthesis of chiral intermediates for pharmaceuticals. Curr Org Chem.

[B4] Straathof AJJ, Panke S, Schmid A (2002). The production of fine chemicals by biotransformations. Curr Opinion Biotechnol.

[B5] Alcalde M, Ferrer M, Plou FJ, Ballesteros A (2006). Environmental biocatalysis: from remediation with enzymes to novel green processes. Trends Biotechnol.

[B6] Sheldon RA, van Rantwijk F (2004). Biocatalysis for sustainable organic synthesis. Aust J Chem.

[B7] Domínguez de María P, Sánchez-Montero JM, Sinisterra JV, Alcántara AR (2006). Understanding *Candida rugosa *lipases: An overview. Biotechnol Adv.

[B8] Reetz MT, Jaeger K-E (1998). Overexpression, immobilization and biotechnological application of *Pseudomonas lipases*. Chem Phys Lipids.

[B9] Cao L (2005). Introduction: Immobilised enzymes: Past, Present and Prospects. Carrier-bound Immobilized Enzymes: Principles, Application and Design.

[B10] Cao L (2005). Immobilised enzymes: science or art?. Curr Opin Chem Biol.

[B11] Mateo C, Palomo JM, Fernandez-Lorente G, Guisan JM, Fernandez-Lafuente R (2007). Improvement of enzyme activity, stability and selectivity via immobilization techniques. Enz Microb Technol.

[B12] López-Serrano P, Cao L, Van Rantwijk F, Sheldon RA (2002). Cross-linked enzyme aggregates with enhanced activity: Application to lipases. Biotechnol Lett.

[B13] Amotz S (1987). Method for production of an immobilized enzyme preparation by means of a crosslinking agent.

[B14] Habeeb AF (1967). Preparation of enzymically active, water-insoluble derivatives of trypsin. Arch Biochem Biophys.

[B15] Jansen EF, Tomimatsu Y, Olson AC (1971). Cross-linking of – chymotrypsin and other proteins by reaction with glutaraldehyde. Arch Biochem Biophys.

[B16] Margolin AL (1996). Novel crystalline catalysts. Trends Biotechnol.

[B17] Brady D, Steenkamp L, Skein E, Chaplin JA, Reddy S (2004). Optimisation of the enantioselective biocatalytic hydrolysis of naproxen ethyl ester using ChiroCLEC-CR. Enz Microb Technol.

[B18] Cao L, van Langen L, Sheldon RA (2003). Immobilised enzymes: carrier-bound or carrier-free?. Curr Opin Biotechnol.

[B19] Moolman FS, Brady D, Sewlall AS, Rolfes H, Jordaan J (2005). Stabilization of Enzymes. Int Pat Appl.

[B20] Aloulou A, Rodriguez JA, Fernandez S, van Oosterhout D, Puccinelli D, Carrie're F (2006). Exploring the specific features of interfacial enzymology based on lipase studies. Biochim Biophy Acta.

[B21] RCSB Protein Data Bank. http://www.rcsb.org/pdb/home/home.do.

[B22] DeLuca T, Kaszuba M, Mattison K (2006). Optimizing silicone emulsion stability using zeta potential. American Laboratory News.

[B23] Cao L, Jeoffrey E (2003). Crosslinked enzyme aggregates and crosslinking agent therefore. US Pat Appl.

[B24] Pencreac'h G, Baratti JC (2001). Comparison of hydrolytic activity in water and heptane for thirty-two commercial lipase preparations. Enz Microb Technol.

[B25] Chaplin JA, Gardiner NS, Mitra RK, Parkinson CJ, Portwig M, Dickson MD, Brady D, Marais SF, Reddy S (2002). Int Pat Appl.

[B26] Chen J, Zhang J, Han B, Li Z, Li J, Feng X (2006). Synthesis of cross-linked enzyme aggregates (CLEAs) in CO_2_-expanded micellar solutions. Coll Surface.

[B27] Chen J, Dickinson E (1995). Protein/surfactant interfacial interactions Part 3. Competitive adsorption of protein + surfactant in emulsions. Coll Surfaces A.

[B28] Grochulski P, Li Y, Schrag JD, Bouthillier F, Smith P, Harrison D, Rubin B, Cygler M (1993). Insights into interfacial activation from an open structure of *Candida rugosa *lipase. J Biol Chem.

[B29] Vorderwülbecke T, Kieslich K, Erdmann H (1992). Comparison of lipases by different assays. Enz Microb Technol.

[B30] Helistö P, Korpela T (1998). Effects of detergents on activity of microbial lipases as measured by the nitrophenyl alkanoate esters method. Enz Microb Technol.

[B31] Jordaan J, Leukes WD (2003). Isolation of a novel laccase with DMAB and MBTH oxidative coupling activity from a mesophilic white rot fungus. Enz Microb Technol.

[B32] Jordaan J, Pletschke BI, Leukes WD (2004). Purifications and partial characterisation of a novel laccase from an unidentified basidiomycete. Enz Microb Technol.

[B33] Steck TL (1972). Cross-linking the major proteins of the isolated erythrocyte. J Mol Biol.

